# Editorial: Sex and Gender Differences in Tinnitus

**DOI:** 10.3389/fnins.2022.844267

**Published:** 2022-02-15

**Authors:** Christopher R. Cederroth, Winfried Schlee

**Affiliations:** ^1^Section of Experimental Audiology, Department of Physiology and Pharmacology, Karolinska Institutet, Stockholm, Sweden; ^2^National Institute for Health Research (NIHR) Nottingham Biomedical Research Centre, Nottingham University Hospitals NHS Trust, Nottingham, United Kingdom; ^3^Hearing Sciences, Division of Clinical Neuroscience, School of Medicine, University of Nottingham, Nottingham, United Kingdom; ^4^Department of Psychiatry and Psychotherapie, University of Regensburg, Regensburg, Germany

**Keywords:** hearing loss, tinnitus, sex, gender, treatment, health

Chronic tinnitus is a common condition that affects nearly 15% of the population. It is a complex disorder caused by a combination of genetic and environmental factors [see e.g., Frontiers Research Topic “*Toward an understanding of tinnitus heterogeneity*” (Cederroth et al., 2019)]. Even though much research progress has been made in recent years, the understanding of the underlying biological mechanisms are still unclear. Recently, some evidence pointed toward greater psychological burden (e.g., increased stress and anxiety) in women when compared to men (Schlee et al., 2017). This guided additional research evaluating the relationships between tinnitus and suicide attempts, whereby women with severe tinnitus have greater odds of suicide attempts, whereas this is not found in men (Lugo et al., 2019). Interestingly, analysis of medical registry data showed that for women that had been diagnosed for tinnitus (i.e., had consulted medical support from specialty care), the association with suicide attempts was no longer seen. This study highlighted that medical care, in spite of the lack of recognized medical treatment, also could impact on the well-being of tinnitus patients. Whether this medical treatment effect is sex or gender specific is however unclear. Other studies have also started evidencing a sex-specific contribution of genetics on various tinnitus traits such as laterality (Maas et al., 2017), or severity (Trpchevska et al., 2020).

Indeed, sex (or gender) as a Biological Variable (SABV) has seldom been investigated in tinnitus research, although its impact on hearing (a known risk factor of tinnitus) is rather well established. Under “sex” we understand the biological classification encoded in the DNA. Under “gender” we understand the respective social roles, behavior and expressions. The aim of this Research Topic was to encourage research in this area. A short summary of the published articles is given below.

In the paper by Basso et al., a sample of 7,615 tinnitus subjects from the general Swedish population was analyzed to identify factors that are associated with bothersome tinnitus, compared to non-bothersome tinnitus. While no gender differences were found for the majority of the analyzed factors, a difference was found in women with bothersome tinnitus that more often reported cardiovascular disease, thyroid disease, epilepsy, fibromyalgia, and burnout. Among men, bothersome tinnitus was more often related to alcohol consumption, Ménière's disease, anxiety syndrome, and panic disorders. The direction of these relationships remain to be assessed.

The authors Niemann et al. also investigated gender effects on a large sample of 1,628 chronic tinnitus patients from the Tinnitus Center Charité. They found that female patients had lower mental and physical health than males, as inferred from the Short Form-9 Health Survey (SF8), likely due to higher levels of tinnitus-related stress. This is in line with previous work from Schlee et al. (2017) and a study from Fioretti et al. in this Research Topic Indeed, analysis of the Perceived Stress Questionnaire (PSQ) revealed that the increased stress-reporting by female patients was related to an increase in worries, tensions and demand, something that was also reflected in the general depression scale, where women scored significantly higher than men. Analysis of the subscales of the tinnitus questionnaire (TQ) revealed that the increased tinnitus-distress in women was mainly due to increased sleep disturbances, difficulties with auditory perception, and somatic complaints, consistent with the study from Edvall et al. on temporomandibular complaints in tinnitus subjects.

Van der Wal et al. analyzed the treatment outcome of 316 tinnitus patients that completed multiple and combinatorial treatments at the Antwerp University Hospital and found an important contribution of gender on the outcome:

- female patients benefit more from high-definition transcranial direct current stimulation (HDtDCS) treatment than men
- female patients benefit more from orofacial therapy than men
- male participants benefit more from the combinational treatment of tinnitus retraining therapy (TRT) + cognitive behavioral therapy (CBT) than female.

These findings are also in line with other studies showing that females benefit more from treatments with transcranial magnetic stimulation (De Ridder et al., 2007) or acoustic stimulation (Partyka et al., 2021).

The present Research Topic highlights the importance of considering sex and gender effects in tinnitus research. There is growing evidence that sex impacts on the psychological burden, and potentially the mechanisms leading to greater tinnitus severity in particular in women, similar to the therapeutic perspective, where they appear more responsive than men. Not only it is important to include sex or gender as a variable in studies, but it is also important to perform sex/gender stratified analyses, as these may also reveal other influences. A recent review provides evidence on how sex and gender are both powerful risk factors for almost every disease, and how they affect every organ (Mauvais-Jarvis et al., 2020). This topic is a call for a greater attention toward the inclusion of sex/gender in tinnitus studies, as this may lead to innovative research and a better understanding of the disorder. New tools such the IGAR^1^ (Integrating Gender Analysis into Research) provide excellent guidance and recommendations for study designs and funding applications.

## Author Contributions

CC and WS contributed equally to the conception and writing of the manuscript. Both authors contributed to the article and approved the submitted version.

## Funding

This work was supported by GNP-182. CC was supported by the UK National Institute for Health Research (NIHR) Biomedical Research Centre.

## Author Disclaimer

CC was supported by the UK National Institute for Health Research (NIHR) Biomedical Research Centre but the views expressed herein are his own and do not represent those of NIHR nor the UK Department of Health and Social Care.

## Conflict of Interest

The authors declare that the research was conducted in the absence of any commercial or financial relationships that could be construed as a potential conflict of interest.

## Publisher's Note

All claims expressed in this article are solely those of the authors and do not necessarily represent those of their affiliated organizations, or those of the publisher, the editors and the reviewers. Any product that may be evaluated in this article, or claim that may be made by its manufacturer, is not guaranteed or endorsed by the publisher.

## Footnotes

^1^ http://igar-tool.gender-net.eu/en/key-guidelines-0
